# Pathways to Mental Well-Being in Young Carers: The Role of Benefit Finding, Coping, Helplessness, and Caring Tasks

**DOI:** 10.1007/s10964-021-01478-0

**Published:** 2021-07-19

**Authors:** Hannah Wepf, Stephen Joseph, Agnes Leu

**Affiliations:** 1 Department of Psychology, University of Zurich, Zurich, Switzerland; 2 Careum School of Health, Kalaidos University of Applied Sciences, Zurich, Switzerland; 3 School of Education, University of Nottingham, Nottingham, UK; 4 Institute for Biomedical Ethics, University of Basel, Basel, Switzerland

**Keywords:** Young carers, Mental health, Stress, Caregiving, Stress-related growth, Resilience

## Abstract

Although prior research has shown that young carers may perceive benefits from their challenging situation, it is unclear how and when benefit finding leads to better mental health. This study examines pathways through which benefit finding may influence mental well-being. Self-reported data were obtained from 601 adolescents aged 15–21 (*M*_age_ = 17.87, 71.9% female) who provided care for a close person with physical or mental health problems. Benefit finding was associated with better mental well-being directly as well as indirectly via better coping and lower helplessness. These findings were similar across young carers with different caring task profiles, except for a few differences regarding social/emotional and instrumental care. The study suggests that benefit finding could promote coping skills and mental well-being in adolescent young carers with implications for the design of future research on interventions with young carers.

## Introduction

A substantial number of youth provide care for family members or close friends with health-related problems (e.g., a disability, mental or physical illness, or frailty; for a review, see Leu and Becker 2019). Their caring tasks can be time-consuming. A recent study found that adolescents and young adults with caring roles spent about 3–5 h helping each day (Haugland et al. 2019). Due to their responsibilities, these young carers’ day-to-day lives can be stressful (e.g., Ali et al. 2012), with potential risks to their own mental health (e.g., Dharampal and Ani 2020). However, not all young carers experience adverse impacts on their emotional well-being (e.g., Svanberg et al. 2010). The relation between caring and mental health is complex, and research still needs to unravel which factors help or hinder young carers’ development and well-being (for a review, see Joseph et al. 2020). Benefit finding, which broadly refers to deriving benefits from experienced difficulties, appears to be a key factor that may determine positive mental health outcomes in young carers (e.g., Cassidy et al. 2014a). Yet, previous studies have not established how and when benefit finding is linked to mental well-being in young carers, and whether benefit finding needs to be in relation to the caring events themselves or life stressors more generally. This study aimed to address this gap by examining pathways through which benefit finding in response to general life stress may lead to mental well-being in young carers aged 15–21 years.

In one of the first studies to investigate benefit finding in young carers, 124 young carers (aged 8–21 years) completed the positive outcomes index of the Positive and Negative Outcomes of Caring instrument (PANOC-YC20), which was found to be negatively associated with depressive symptoms (Joseph et al. 2009). Applying the Benefit Finding in Child Caregivers Scale (BFCCS, Cassidy and Giles 2013), a further study of 442 young carers (aged 12–16 years) found associations between benefit finding scores and measures of positive and negative mental health outcomes (Cassidy et al. 2014a). More recently, two studies examining youth of parents with a serious health problem found caring-related benefit finding was associated with positive outcomes including prosocial behavior, positive affect, as well as life and family satisfaction among 428 youth (aged 9–20 years; Pakenham and Cox 2018) and with psychological well-being among 246 children and adolescents (aged 8–18 years; Kallander et al. 2018). Taken together, this small but growing body of literature recognizes that young carers commonly perceive positive aspects of their situation, and the extent of such positive perceptions is likely to be related to better mental well-being.

From a theoretical perspective, young carers’ benefit finding may be directly associated with mental well-being. However, it is likely that more complex mechanisms linking benefit finding and mental well-being are also in operation. Empirical evidence supports the notion of a stress and coping model that considers benefit finding as an important resource to understand the variability in the outcome of caring on youth (Cassidy et al. 2014a). According to stress and coping theory, individuals’ responses to potentially stressful situations vary depending on their appraisal of the event and their resources to handle the requirements (Lazarus and Folkman 1984).

### Proposed Pathways From Benefit Finding

**Fig. 1:**
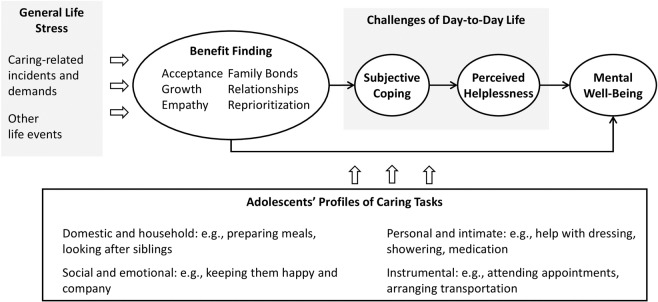
Conceptual model depicting proposed pathways from benefit finding in response to general life stress on mental well-being in adolescents with caring responsibilities

Evidence suggests that benefit finding can lead to increased psychological and social resources and may thus alter the way individuals respond to future challenges (Bower et al. 2009). A potential explanation of such a relationship may be found in the literature on the role of self-reflection in the process of stress-related growth. It has been proposed that reflective practices may help individuals develop insight into already-present capacities and regulatory flexibility and thus improve their strategies to cope with future difficulties (Crane et al. 2019). As illustrated in Fig. 1, this study proposes that the positive relationship between benefit finding and mental well-being outcomes is partly explained in terms of a mechanism that buffers feelings of helplessness thanks to enhanced coping abilities. The possibility that young carers’ experiences with past stressors can improve their subsequent coping has been discussed in the young carers literature (e.g., Greene et al. 2017), but this pathway has not yet been tested in samples of adolescents with caring roles.

Nevertheless, research provides some preliminary support for the assumption that benefit finding is involved in important stress-coping mechanisms among young carers. For instance, caring-related benefits were associated with the use of adaptive coping strategies (i.e., acceptance, problem solving, seeking social support, Pakenham et al. 2007) and social skills (Kallander et al. 2018) among children and adolescents who have a parent with a serious health problem. Quantitative research on experiences of stress in young carers is underdeveloped. Consequently, little is known about the relationships between benefit finding and indicators of perceived stress or stress responses. While two studies showed that caring-related benefit finding is associated with lower appraisals of caring role stress among young carers (Cassidy et al. 2014a; Cassidy and Giles 2013), this finding was not supported by a study among children and adolescents affected by parental health problems (Pakenham et al. 2007). It remains unknown, to date, how benefit finding is related to experiences of stress in young carers’ lives that are not or indirectly related to their caring roles.

### Caring Tasks as Moderating Factors

Different caring demands may provoke different types of stressors and thus require distinct coping strategies. Hence, associations between benefit finding, coping, helplessness, and mental well-being are likely to vary depending on the caring tasks in which young carers may be involved. The young carers literature commonly describes four different caring domains: *domestic and household care*, *personal and intimate care*, *social and emotional care*, and *instrumental care* (e.g., Ireland and Pakenham 2010; see Fig. 1 for examples of tasks). Previous research has indicated that caring tasks from these different domains may have different impacts on youth adjustment. Two studies found social/emotional care to be the only type of caring tasks that are positively associated with youth adjustment (i.e., health and prosocial behavior: Ireland and Pakenham 2010; and health-related quality of life: Landi et al. 2021). Another study found no association with social/emotional caring tasks, but suggested that more tasks from the personal/intimate care domain are negatively associated with psychological well-being among children who have a parent with health problems (Kallander et al. 2018). Consequently, the types of caring tasks may be understood as moderating factors of pathways between benefit finding and mental well-being (see Fig. 1).

### Measures of Benefit Finding in Young Carers: Boundaries and Opportunities

Previous research on benefit finding in young carers applied instruments that capture experiences that are directly related to young people’s caring roles (e.g., Pakenham and Cox 2018; Cassidy et al. 2014a). These studies advanced the understanding of the experiences related to a caring role, but they also have limitations for research and practice. First, the impact of caring cannot be extracted from the numerous other experiences young carers have in their lives (e.g., McGibbon 2021). Previous research neglected the interrelatedness of stressors that young carers face, exemplified by strain resulting from the accumulation of tasks and requirements of different life domains such as caring, school, work and social life (e.g., Stamatopoulos 2018). Moreover, assessing caring-related benefit finding may also be a problematic approach for the design of interventions, for instance among those who may not self-identify as carers (e.g., because it seems normal to them; Smyth et al. 2011) or when youth do not want to disclose their caring roles (e.g., McGibbon 2021). Therefore, a more general view on benefit finding is needed to advance the understanding of young carers’ well-being and development. This can be achieved by considering more general perceptions and sources of stress in young people’s past and current life (related or unrelated to caring). Second, the assessment of benefit finding in young carers thus far relied on unidimensional scales. The types of benefits youth perceive could be differently related to mental well-being, however. Accordingly, different dimensions of benefit finding should be considered.

This study took the opportunity to move forward the measurement of benefit finding in young carers by addressing these boundaries. Specifically, the General Benefit Finding Scale (GBFS; Cassidy et al. 2014b) was used. In doing so, benefit finding was conceptualized and measured as adolescents’ overall perceptions of benefits in response to general life stress including life events that are related or unrelated to caring (see Fig. 1). This measure also enabled the consideration of the six benefit finding dimensions, i.e., acceptance, family bonds, relationships, growth, empathy, and reprioritization.

## Current Study

The present study posed the question of how and when deriving benefits from stressful experiences is associated with mental well-being in young carers. Structural equation modeling (SEM) was used to test a cross-sectional model of the associations between benefit finding in response to general life stress and current mental well-being. First, a direct path (i.e., benefit finding → mental well-being) and an indirect path (i.e., benefit finding → subjective coping → perceived helplessness → mental well-being) were proposed to test if the association between benefit finding and mental well-being is mediated by higher subjective coping resources and lower feelings of helplessness. Second, it was predicted that regression paths would differ across subgroups of adolescents with a low or high frequency of caring tasks within the four domains (i.e., domestic/household, personal/intimate, social/emotional, and instrumental care). Finally, the potentially differing roles of benefit finding in terms of its subdimensions were explored.

## Method

### Participants and Procedures

Data were derived from a survey completed by 2525 adolescents (ranging from 15 to 21 years) recruited through different educational institutions in the German-speaking part of Switzerland during 2018 and 2019 (see also Wepf et al. 2021). Informed consent was obtained from all participants via an online form and the institutional Ethics Committee from the Faculty of Arts and Social Sciences of the University of Zurich approved the project procedures. All participants who indicated that they knew a close person with a health-related need for support in daily life were asked about caring tasks during the six months preceding the day of data collection. The frequency of caring tasks was rated for each of four caring domains (i.e., domestic/household, personal/intimate, social/emotional, and instrumental) using 5-point rating scales: 1 = *never*, 2 = *rarely*, 3 = *now and then*, 4 = *often*, 5 = *very often* (for more details see “Frequency of caring tasks” under measures below). A reported frequency of 3, 4, or 5 in at least one of the four domains of care was chosen as the criterion for a current young carer. This provided a subset of 601 young carers.

The young carers sample had a mean age of 17.87 years (*SD* = 1.53). Participants identified as female (71.9%), male (27.6%), or other (0.5%). Most of the participants (72.9%) reported being Swiss. The educational institutions participants attended were a mix of vocational training schools (*n* = 493), vocational training companies (*n* = 47), high schools (*n* = 41), and transitional options (*n* = 20). Regarding their caring roles, 50.1% of the participants reported that the health problem of the care recipient was mental/cognitive difficulty; 30.1% reported a physical difficulty, and 19.8% reported a combination of physical and mental/cognitive difficulties. Most participants (68.9%) cared for a family member (parent: 27.8%, grandparent: 21.0%, sibling: 11.5%, another family: 8.7%). The remaining cared for a close friend (13.5%), spouse or boyfriend/girlfriend (7.7%), or another person they felt close and committed to (10.0%). About half of the participants (47.9%) lived with the care recipient (35.8% *all of the time*, 12.1% *partly*). On average, participants started to provide care at the age of 13, which meant that at the time of data collection they had on average been caring for 4.47 years (*SD* = 4.13). About one third (33.4%) reported that, besides the described main care recipient, there were one or multiple persons close to them who also needed care because of health problems (14.0% one additional person, 19.4% multiple additional persons).

### Measures

All items were self-reported and part of a broader online questionnaire on the psychosocial well-being of adolescents administered in German, the official language of the study locations.

#### Mental well-being

Mental well-being was assessed using the 14-item Warwick–Edinburgh Mental Well-being Scale (WEMWBS, Lang and Bachinger 2017; Tennant et al. 2007) but only the seven items belonging to the short version of the scale (i.e., SWEMWBS; Stewart-Brown et al. 2009) were used in the final analyses in this study (e.g., “I’ve been feeling relaxed”, “I’ve been thinking clearly”, “I’ve been feeling close to other people”). Items were rated on a 5-point scale (0 = *none of the time*, 4 = *all of the time*). The standardized Cronbach’s alpha for the SWEMWBS mean score in this current study was *α* = 0.80.

#### Benefit finding

The 28-item General Benefit Finding Scale (GBFS, Cassidy et al. 2014b) was used to measure adolescents’ subjective experience of positive changes in response to lifetime adversity. The introductory text of the questionnaire asked participants to consider difficult times they had had in their life and to respond to the scale in relation to how they felt living through those difficult times by indicating on a 5-point scale how much each item was true for them (1 = *not true at all*, 5 = *absolutely true*). The GBFS can be used as an overall score (*α* = 0.90) but it also has six subscales, i.e., acceptance (5 items, e.g., “Led me to be more accepting of things”, *α* = 0.76), family bonds (4 items, e.g., “Brought my family closer together”, *α* = 0.78), growth (6 items, e.g., “Made me a more effective person”, *α* = 0.84), relationships (4 items, e.g., “Helped me become more aware of the support available from others”, *α* = 0.70), empathy (5 items, e.g., “Made me more compassionate to those in similar situations”, *α* = 0.74), and reprioritization (4 items, e.g., “Led me to place less emphasis on material things”, *α* = 0.65). Items used in this study were translated into German by two independent researchers and then carefully discussed to find a consensus for the final wording appropriate for the Swiss adolescents in this study.

#### Perceived helplessness

As an indicator of adolescents’ stress responses, the perceived helplessness scale, i.e., a subscale of the 10-item Perceived Stress Scale (PSS-10, Cohen et al. 1983; Klein et al. 2016), was used. The six items referred to the past month (e.g., “How often have you felt difficulties were piling up so high that you could not overcome them?”) and were scored on a 5-point scale (0 = *never*, 4 = *very often*). The standardized Cronbach’s alpha in this study was *α* = 0.82.^1^

#### Subjective coping

Adolescents’ perception of their own coping strategies was measured by two single items that serve as additional information in the Incope-2 questionnaire which is a validated Swiss questionnaire that measures individual coping strategies (Bodenmann 2000). The two items cover participants’ efficacy of and satisfaction with their individual coping (i.e., “I am satisfied with how I cope with stress”. and “My way of dealing with stress is usually effective.”) and were rated on 0 (*never*) to 4 (*very often*) scales. The standardized Cronbach’s alpha across the two items was *α* = 0.83.

#### Frequency of caring tasks

The domains of caring tasks covered: domestic/household care (e.g., cleaning, grocery shopping, cooking, looking after siblings, etc.), personal/intimate care (e.g., help with eating, washing or toileting, help with medication, etc.), social/emotional care (e.g., cheering up, keeping company, make sure the person is safe, etc.), and instrumental care (e.g., coordination of appointments, paying bills, doing phone calls, organizing transportation, etc.). For each of the four domains, the participants rated on a 5-point scale (1 = *never*, 2 = *rarely*, 3 = *now and then*, 4 = *often*, 5 = *very often*) how often they had carried out these caring tasks during the past six months. (As described in the Participants section, these same four items served as information to identify young carers from the overall sample of adolescents.) For multi-group analyses, the median of each of the four scales was used as a cut-off point for splitting the overall data into two comparable-sized subgroups.

The median of the frequency rating in the domestic/household care domain was 3 (*low domestic/household care*: *n* = 247, 41.1%, frequency rating <3, i.e., “never” or “rarely”; *high domestic/household care*: *n* = 354, 58.9%, frequency rating ≥3, i.e., “now and then”, “often”, or “very often”). The median of the frequency rating in the personal/intimate care domain was 2 (*low persona/intimate care*: *n* = 287, 47.8%, frequency rating <2, i.e., “never”; *high personal/intimate care*: *n* = 314, 52.2%, frequency rating ≥2, i.e., “rarely”, “now and then”, “often”, or “very often”). The median of the frequency rating in the social/emotional care domain was 4 (*low social/emotional care*: *n* = 232, 38.6% frequency rating <4, i.e., “never”, “rarely”, or “now and then”; *high social/emotional care*: *n* = 369, 61.4%, frequency rating ≥4, i.e., “often” or “very often”). The median of the frequency rating in the instrumental care domain was 2 (*low instrumental care*: *n* = 237, 39.4%, frequency rating <2, i.e., “never”; *high instrumental care*: *n* = 364, 60.6%, frequency rating ≥ 2, i.e., “rarely”, “now and then”, “often”, or “very often”).

### Analytical Strategy

Incomplete surveys in terms of dropout were excluded case-wise (*n* = 21) and the survey did not allow respondents to skip questions. Therefore, the analysis dataset had no missing values. All analyses were performed in R (Version 4.0.0; R Core Team 2020) with RStudio (Version 1.4.1103; RStudio Team 2021). First, the measurement model was tested using the confirmatory factor analyses function *cfa*, in the *lavaan* package (Version 0.6–5; Rosseel 2012) and *s*econd, the proposed model was tested using the SEM function *sem* in the same package. Model fit was assessed using the following global indices: chi-square test statistic (χ^2^) with degrees of freedom (df), comparative fit index (CFI), standardized root mean square residual (SRMR), and root mean square error of approximation (RMSEA). Acceptable fit between the conceptual model and the observed data was indicated when CFI ≥ 0.90, SRMR ≤ 0.08, and RMSEA ≤ 0.08. To examine significance of the proposed indirect effects within the model, 10,000 bootstrap samples with 95% bias-corrected confidence intervals were estimated (Preacher and Hayes 2008). Accordingly, the confidence intervals of unstandardized effects that did not pass through zero indicated significance of the corresponding indirect path. Third, to assess whether the model pathways were equivalent across subgroups, multiple-sample SEM analyses were conducted.

Before testing for structural invariance across the groups, invariance of the measurement part of the model was assessed (Kline 2016). Then, the hypotheses of differences between the groups concerning the regressions paths were tested by stepwise imposing cross-group equality constraints on the regression estimates of one parameter at the time. If model fit while imposing the equality constraints was poorer than for the more relaxed model (chi-square difference test), it was concluded that there was evidence for group differences on this specific parameter (Kline 2016), and the constraints were eliminated from the model. If the difference between the model fit was not significant, it was concluded that the parameters could stay constrained in the model and the next constraint was added to the model. Since multiple comparisons were conducted, an alpha level of 0.01 was used as the significance level in group comparisons.

## Results

### Measurement Model

**Fig. 2:**
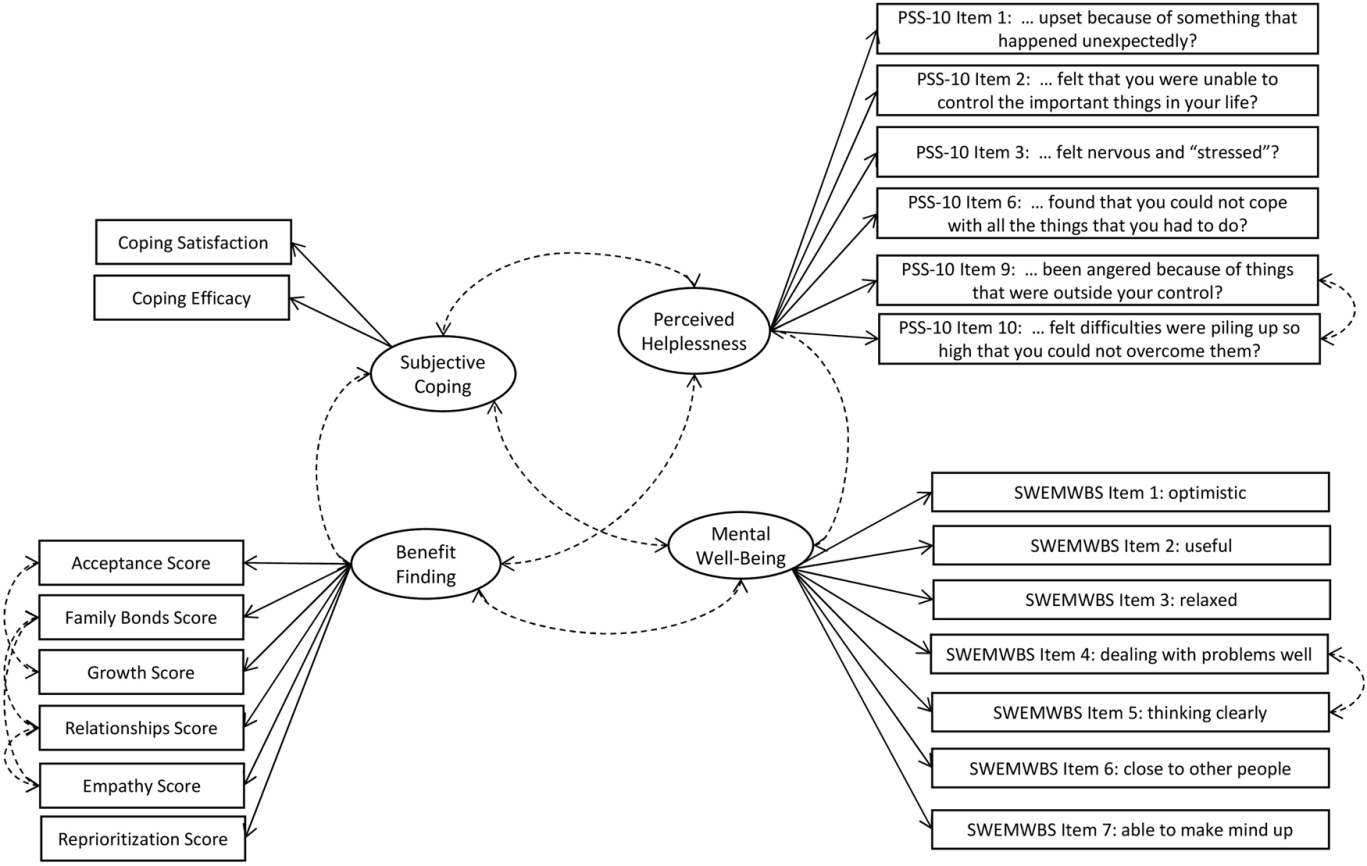
Modified final measurement model

The measurement model using all variables of interest was tested with the overall young carers sample (*n* = 601). This model included the six GBFS subscale mean scores as indicators for “benefit finding”; the two one-item rating scales for assessing satisfaction and effectiveness of individual coping as indicators of “subjective coping”; the six items of the perceived helplessness subscale from the PSS-10 measuring “perceived helplessness” (as a proxy for perceived stress response), and the seven items of the SWEMWBS measuring “mental well-being”. The fit indices for this measurement model were χ^2^ (183) = 693.44, *p* < 0.001, CFI = 0.91, RMSEA = 0.07 (90% CI 0.06, 0.07), SRMR = 0.06. Inspection of residuals as well as modification indices in combination with theoretical considerations suggested correlated error terms between the GBFS subscales relationships with empathy, acceptance with growth, and family bonds with empathy as well as relationships; the PSS-10 item 9 and 10, and the SWEMSBS items 4 and 5. The six modified measurement models were tested sequentially by adding one error term at the time. Results indicated that regarding each of the re-estimated models the fit improved significantly. Thus, the sixth modified measurement model with the following fit indices was kept: χ^2^ (177) = 469.22, *p* < 0.001, CFI = 0.95, RMSEA = 0.05, (90% CI 0.05, 0.06), SRMR = 0.06. Figure 2 shows the final measurement model. Further details on the measurement model are reported in Tables 1 and 2.

**Table 1:** Final measurement model of the hypothesized model

Latent variables and corresponding observed indicators	Stand. Loadings	*M*	SD	*S*	*K*	*α*
Benefit finding						0.90
Acceptance score	0.83	2.54	0.78	−0.40	0.14	
Family bonds score	0.58	2.59	0.91	−0.54	−0.06	
Growth score	0.85	2.54	0.81	−0.57	0.12	
Relationships score	0.80	2.64	0.81	−0.59	0.32	
Empathy score	0.64	2.65	0.81	−0.50	0.08	
Reprioritization score	0.86	2.44	0.79	−0.31	0.06	
Perceived helplessness						0.82
PSS-10 Item 1: … upset because of something that happened unexpectedly?	0.50	2.21	1.06	−0.10	−0.66	
PSS-10 Item 2: … felt that you were unable to control the important things in your life?	0.75	2.23	1.15	−0.20	−0.77	
PSS-10 Item 3: … felt nervous and “stressed”?	0.69	3.00	0.99	−0.77	−0.10	
PSS-10 Item 6: … found that you could not cope with all the things that you had to do?	0.73	2.12	1.01	−0.10	−0.36	
PSS-10 Item 9: … been angered because of things that were outside your control?	0.47	2.35	1.09	−0.26	−0.56	
PSS-10 Item 10: … felt difficulties were piling up so high that you could not overcome them?	0.73	2.30	1.19	−0.22	−0.82	
Subjective coping						0.83
My way of dealing with stress is usually effective.	0.90	2.03	1.12	−0.06	−0.69	
I am satisfied with how I cope with stress.	0.78	2.09	1.08	−0.15	−0.49	
Mental well-being						0.80
SWEMWBS Item 1: … feeling optimistic about the future	0.58	3.53	0.97	−0.39	−0.29	
SWEMWBS Item 2: … feeling useful	0.68	3.60	0.92	−0.56	0.02	
SWEMWBS Item 3: … feeling relaxed	0.62	2.86	1.03	0.22	−0.56	
SWEMWBS Item 4: … dealing with problems well	0.66	3.45	0.97	−0.47	−0.12	
SWEMWBS Item 5: … thinking clearly	0.65	3.44	0.99	−0.25	−0.52	
SWEMWBS Item 6: … feeling close to other people	0.43	3.63	1.01	−0.54	−0.21	
SWEMWBS Item 7: … able to make up my own mind about things	0.61	3.95	0.91	−0.63	0.01	

*N* = 601. The error terms were correlated between GBFS subscales scores relationships and empathy, family bonds and empathy, acceptance and growth, and relationships and family bonds; as well as the PSS-10 item 9 and 10, and SWEMSBS items 4 and 5

**Table 2:** Correlations for latent variables

Variable	1	2	3	4
1. Benefit Finding	–			
2. Perceived Helplessness	0.01	–		
3. Subjective Coping	0.36	−0.40	–	
4. Mental Well-being	0.46	−0.61	0.54	–

*N* = 601

### Structural Model

**Fig. 3:**
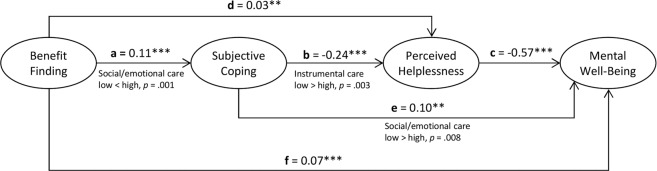
Structural equation model depicting the direct and indirect effects of benefit finding on mental well-being in young carers including unstandardized coefficients for the overall Sample (*n* = 601, *p < 0.05 **p < 0.01 *** *p* < 0.001) and notes regarding differences by frequency of caring tasks (only shown if *p* < 0.01)

The full structural model was specified with direct and indirect paths including the predictions as well as the two additional indirect paths that would be possible (i.e., benefit finding → subjective coping resp. perceived helplessness → mental well-being). The original model demonstrated an acceptable fit to the young carers data: χ^2^ (177) = 469.22, *p* < 0.001, CFI = 0.95, RMSEA = 0.05 (90% CI 0.05, 0.06), SRMR = 0.06. Figure 3 presents the model including unstandardized estimates for the paths. Table 3 shows the standardized estimates for all direct and indirect effects including 95% bias-corrected confidence intervals based on bootstrapping with 10,000 samples.

**Table 3:** Unstandardized and standardized direct and indirect effects for the model predicting mental well-being in the overall sample (*N* = 601)

	Unst. estimate	*SE*	*p*	95% CI	Stand. estimate
Benefit finding to mental well-being					
Total effect	0.08	0.01	<0.001	0.062, 0.104	0.46
Direct effect	0.07	0.01	<0.001	0.051, 0.093	0.40
Total indirect effects	0.01	0.01	0.130	−0.003, 0.062	0.06
bf → coping → stress → mwb	0.02	0.00	<0.001	0.010, 0.025	0.09
bf → coping → mwb	0.01	0.00	0.005	0.004, 0.020	0.07
bf → stress → mwb	−0.02	0.01	0.006	−0.030, −0.006	−0.09
Coping to mental well-being					
Total effect	0.24	0.04	<0.001	0.176, 0.316	0.43
Direct effect	0.10	0.04	0.005	0.035, 0.177	0.18
Indirect effect					
coping → stress → mwb	0.14	0.03	<0.001	0.096, 0.196	0.25
Stress to mental well-being					
Direct effect	−0.57	0.08	<0.001	−0.743, −0.438	−0.54

CI = 95% bias corrected confidence intervals based on 10,000 bootstrap samples. Confidence intervals of unstandardized effects that do not pass through zero indicate significance

*mwb* mental well-being, *bf* benefit finding, *coping* subjective coping, *stress* perceived helplessness

Consistent with the prediction, there was a direct effect of benefit finding on mental well-being (*b* = 0.07). The results also indicated an indirect effect of benefit finding over subjective coping and perceived helplessness on mental well-being (path: a*b*c, *b* = 0.02, 95% CI: 0.010, 0.025). Furthermore, the two additional indirect effects in the model were examined. The indirect effect of benefit finding on mental well-being over subjective coping (path: a*e, *b* = 0.01, 95% CI: 0.004, 0.020) as well as over perceived helplessness (path: d*c, *b* = −0.02, 95% CI: −0.030, −0.006) were significant. However, the effect over perceived helplessness was negative. The total indirect effect of benefit finding on mental well-being in the model was therefore canceled out and was not significant (*b* = 0.01, 95% CI: −0.003, 0.062). The total effect of the benefit finding was *b* = 0.08 and significant (95% CI: 0.062, 0.104). The full structural model (Fig. 3) explained 61% of the variance in mental well-being.

### Multi-Group Analyses

As a next step, four sets of multi-group comparisons were conducted to test for differences regarding the positive effects of benefit finding on mental well-being between young carers with a low relative to high frequency of tasks regarding each caring domain. As described in the Analytical Strategy section, first it was determined whether the measurement was invariant in terms of factor loadings and intercepts (strong invariance), which would mean that comparisons of model paths between groups are allowed (Kline 2016).

The measurement model (as used above) in which loadings and intercepts were constrained across the two groups was not significantly different from the same model without these constraints (all *p* > 0.05 except for social/emotional care with *p* > 0.01), which indicated that comparisons between groups could be made (domestic/household care: Δχ^2^ = 27.76, Δdf = 34, *p* = 0.766; personal/intimate care: Δχ^2^ = 46.72, Δdf = 34, *p* = 0.072; social/emotional care: Δχ^2^ = 49.18, Δdf = 34, *p* = 0.045; instrumental care: Δχ^2^ = 40.18, Δdf = 34, *p* = 0.215). The structural model (as used above, see Fig. 3) estimating the coefficients with constrained loadings and intercepts, but no further paths equality constraints across groups (i.e., regression paths were estimated separately for the low versus high group and allowed to differ) had acceptable fit indices (domestic/household care: χ^2^ (388) = 740.60, *p* < 0.001, CFI = 0.94, RMSEA = 0.06 (90% CI 0.05, 0.06), SRMR = 0.06; personal/intimate care: χ^2^ (388) = 744.30, *p* < 0.001, CFI = 0.94, RMSEA = 0.06 (90% CI 0.05, 0.06), SRMR = 0.07; social/emotional care: χ^2^ (388) = 750.79, *p* < 0.001, CFI = 0.94, RMSEA = 0.06 (90% CI 0.05, 0.06), SRMR = 0.07; instrumental care: χ^2^ (388) = 700.75, *p* < 0.001, CFI = 0.95, RMSEA = 0.05 (90% CI 0.05, 0.06), SRMR = 0.07). The unstandardized estimates of direct and indirect effects on mental well-being for each group are displayed in Table 4.

**Table 4:** Unstandardized path estimates between models for subgroups of young carers with low and high frequency of caring tasks across four domains

	Model							
	Domestic/household care		Personal/intimate care		Social/emotional care		Instrumental care	
	Low (*n* = 247)	High (*n* = 354)	Low (*n* = 287)	High (*n* = 314)	Low (*n* = 232)	High (*n* = 369)	Low (*n* = 237)	High (*n* = 364)
Benefit finding to mental well-being								
Total effect	0.08***	0.08***	0.09***	0.08***	0.07***	0.09***	0.09***	0.08***
Direct effect	0.06**	0.08***	0.07***	0.08***	0.06***	0.08***	0.07***	0.07***
Total indirect effects	0.02	0.01	0.02	0.00	0.01	0.01	0.02	0.00
bf → coping → stress → mwb	0.02**	0.01**	0.02**	0.02***	**0.01**	0.02***	0.03**	0.01**
bf → coping → mwb	**0.01**	0.01*	0.01*	**0.01**	**0.01**	**0.01**	**0.01**	0.01**
bf → stress → mwb	−**0.01**	−0.02*	−**0.01**	−0.02**	−**0.01**	−0.02*	−**0.02**	−0.02*
Coping to mental well-being								
Total effect	0.28***	0.21***	0.27***	0.20***	0.34***	0.18***	0.25***	0.23***
Direct effect	**0.11**	0.09*	0.13*	**0.07**	0.19**	**0.05**	**0.05**	0.12**
Indirect effect								
coping → stress → mwb	0.17***	0.12***	0.14***	0.13***	0.15***	0.13***	0.20***	0.10***
Stress to mental well-being								
Direct effect	−0.63***	−0.54***	−0.54***	−0.60***	−0.63***	−0.55***	−0.59***	−0.58***
* R*^2^ in mental well-being	0.68	0.56	0.60	0.61	0.64	0.61	0.63	0.60

Loadings and intercepts were constrained across the two groups of comparison. Bold font refers to coefficients that were different from the coefficients in the model in Table 3 in terms of significance (yes or no) or direction

*mwb* mental well-being, *bf* benefit finding, *coping* subjective coping, *stress* perceived helplessness

* *p* < 0.05 ** *p* < 0.01 *** *p* < 0.001

To test for differences regarding the model paths between the groups, paths were sequentially constrained to be equal across groups, and the overall fit of the more constrained model was compared to the previous, less constrained model for reductions in fit. Concerning domestic/household care and personal/intimate care, these comparisons revealed that, despite numerical differences (see Table 4), none of the path coefficients differed significantly between groups. Thus, the relative associations for these relationships were similar in young carer subgroups with a low as compared to a high frequency of these types of caring tasks. Regarding social/emotional care, differences were found for path a (benefit finding → subjective coping; low: *b* = 0.06 and high: *b* = 0.16, Δχ^2^ = 10.88, Δdf = 1, *p* = 0.001) and path e (subjective coping → mental well-being; low: *b* = 0.19 and high: *b* = 0.05, Δχ^2^ = 7.08, Δdf = 1, *p* = 0.008), and with regard to differences between low and high instrumental care for path b (subjective coping → perceived helplessness; low: *b* = −0.34 and high: *b* = −0.18, Δχ^2^ = 8.64, Δdf = 1, *p* = 0.003; see Fig. 3).^2^

### Dimensions of Benefit Finding

Thanks to the use of the GBFS (Cassidy et al. 2014b), a multidimensional measure for assessing benefit finding, analyses could be repeated with each of the six subscales of the benefit finding construct as latent exogenous variables. Due to multicollinearity issues, the analyses were run separately for each of the dimensions. Model fit was acceptable for each of the six subscales. For the subdimension of acceptance, family bonds, relationships, and reprioritization, these exploratory analyses revealed that the results were similar to the model with the overall benefit finding variable (Fig. 3, Table 3) in terms of significance and direction of effects. For growth however, the indirect effect from benefit finding (as measured by the growth items) over perceived helplessness on mental well-being (path d*c) was not significant and, as a consequence, the total indirect effect was positive and significant. In the model based on the empathy subscale, there were no indirect effects of benefit finding (measured by the empathy items) over subjective coping on mental well-being and only the negative indirect effect over perceived helplessness (path d*c, but not a*b*c and a*e) was significant. The fit indices and estimates for each of the six models are displayed in Tables 5 and 6.

**Table 5:** Additional analyses: global fit indices for models of each of the six the benefit finding subscales

Model	*χ*^2^	df	*p*	CFI	RMSEA (95% CI)	SRMR
Acceptance	374.88	162	<0.001	0.95	0.05 (0.04, 0.05)	0.05
Family bonds	366.14	144	<0.001	0.94	0.05 (0.04, 0.06)	0.06
Growth	454.09	181	<0.001	0.94	0.05 (0.04, 0.06)	0.05
Relationships	333.74	144	<0.001	0.95	0.05 (0.04, 0.05)	0.05
Empathy	349.69	162	<0.001	0.95	0.04 (0.04, 0.05)	0.05
Reprioritization	398.53	144	<0.001	0.93	0.05 (0.05, 0.06)	0.06

*N* = 601. The error terms were correlated between the PSS-10 item 9 and 10, and SWEMSBS items 4 and 5

**Table 6:** Additional analyses: unstandardized path estimates for models of each of the six the benefit finding subscales using the overall sample (*N* = 601)

	Model					
	Acceptance	Family bonds	Growth	Relationships	Empathy	Reprioritization
Benefit finding to mental well-being						
Total effect	0.33***	0.16***	0.39***	0.38***	0.12**	0.39***
Direct effect	0.27***	0.15***	0.29***	0.34***	0.17***	0.36***
Total indirect effects	0.06	0.01	0.09**	0.05	−0.05	0.03
bf → coping → stress → mwb	0.08***	0.02*	0.07***	0.05**	**0.01**	0.08**
bf → coping → mwb	0.05*	0.03*	0.05*	0.06**	**0.02**	0.06**
bf → stress → mwb	−0.07**	−**0.04**	−**0.03**	−0.06*	−0.08**	−0.11**
Coping to mental well-being						
Total effect	0.23***	0.28***	0.21***	0.25***	0.28***	0.25***
Direct effect	0.09*	0.17**	0.09*	0.13***	0.17***	0.10**
Indirect effect						
coping → stress → mwb	0.14***	0.11***	0.12***	0.12***	0.12***	0.15***
Stress to mental well-being						
Direct effect	−0.56***	−0.53***	−0.53***	−0.56***	−0.56***	−0.60***
*R*^2^ in mental well-being	0.59	0.52	0.61	0.59	0.52	0.60

Loadings and intercepts were constrained across the two groups of comparison. Bold font refers to coefficients that were different from the overall benefit finding model in Table 3 in terms of significance (yes or no) or direction

*mwb* mental well-being, *bf* benefit finding, *coping* subjective coping, *stress* perceived helplessness

*p < 0.05 **p < 0.01 ***p < 0.001

### Sensitivity Analyses

As a matter of robustness checks, the main analyses were re-run without cases that were flagged for potential response bias (e.g., outliers in terms of the participants responding carelessly or answers exhibiting comprehension problems, *n* = 11). The results were substantively the same as those using the full sample.

## Discussion

To design interventions meeting the needs of youth with caring responsibilities, it is crucial to thoroughly understand the experiences and challenges of these young people as well as factors promoting their well-being. Previous research indicates that young carers who perceive positive aspects of their challenging situation demonstrate better mental health. However, the potential mechanisms between benefit finding and mental well-being are not yet understood. This current study aimed to address this gap and proposed that benefit finding would be associated with mental well-being in adolescent young carers both directly and indirectly over a stress-coping mechanism. Moreover, it was examined whether the associations between benefit finding and mental well-being may differ depending on caring tasks carried out by youth.

### How is Benefit Finding Associated With Mental Well-Being in Young Carers?

The first aim of the study was to test the assumption that benefit finding partly leads to better mental well-being in young carers thanks to increased coping resources which lower feelings of helplessness. A central finding is therefore that the direct association between benefit finding and mental well-being as well as the indirect association through subjective coping and perceived helplessness were statistically significant. These positive associations between benefit finding and mental well-being are consistent with the hypotheses. As such, findings may be interpreted as preliminary evidence for the capacity of benefit finding to buffer stress as a result of more adaptive coping abilities.

The findings also support a small body of previous studies on young carers in which associations between caring-related benefit finding and adjustment outcomes were documented. However, this study extends the knowledge to the specific age group of adolescents and a broader conceptualization of benefit finding. While benefit finding in this study reflects young carers’ general tendency to derive benefits from past difficulties, benefit finding in prior research may rather be understood as a way of coping with ongoing difficulties. This is because benefit finding was measured in response to an ongoing difficult situation (Helgeson et al. 2006; Folkman 2008), namely the caring context. For the interpretation of the current study’s findings, this means that not only perceived benefits that are directly related to caring but also other experiences are relevant to well-being in adolescents.

Rather surprisingly, there was a weak positive association between benefit finding and perceived helplessness. There are several potential explanations for this finding. On the one hand, benefit finding may trigger the perception of stress because a more positive view of challenges including the caring role is also likely to motivate young carers to hold on to their responsibilities and even seek more opportunities to fulfill caring duties (e.g., outside the family; Skovdal and Andreouli 2011). In this sense, it seems plausible that high levels of benefit finding can lead to feelings of being overwhelmed. On the other hand, perceptions of stress including feelings of helplessness may also stimulate young carers’ benefit finding, since the ongoing experience of stress may remind young carers of the past stressors and initiate or strengthen positive self-reflection practices. Thus, an even more complex relationship including feedback loops and bidirectional relationships may be at play which would require longitudinal data to address.

### When is Benefit Finding Associated With Mental Well-Being in Young Carers?

The second aim of the study was to determine the contexts in which benefit finding’s capacity to promote mental well-being would enfold. To this end, it was examined whether path coefficients varied as a function of different types of caring tasks provided. The group comparisons revealed differences regarding social/emotional care. The predicted indirect association of benefit finding and mental well-being through coping and helplessness was statistically significant among young carers providing exceptional levels of social/emotional support (i.e., often or very often as compared to less frequently). Specifically, benefit finding’s association with subjective coping was stronger among this group of young carers, suggesting that providing social/emotional care may be a key driver of benefit findings’ coping-enhancing effect. This finding could provide an explanation for the positive associations between social/emotional care and positive adjustment outcomes in previous studies on young carers (e.g., Landi et al. 2021). Social/emotional care means that young carers spend time close together with the care recipient and involves tasks (e.g., cheering them up or making sure the person is safe) that require a positive and trusted relationship. The findings of the current study may therefore also point to the importance of the carer-care recipient relationship for positive experiences among young carers, as has been highlighted in the literature on adult carers (e.g., Cassidy 2013). Regarding the three other caring domains, the role of benefit finding was largely similar across groups with a high and low frequency of caring tasks.

However, it seems important to note that the association between coping and helplessness was weaker among those young carers who performed instrumental tasks (as compared to never). This evidence could suggest that the burden due to instrumental tasks may overwhelm young carers in the sense that the requirements exceed their resources (e.g., energy, time, knowledge, skills), even if they generally feel positive about their ways of coping with challenges. Supporting this view, it was suggested that caring tasks might reinforce burden due to practical difficulties in balancing caring responsibilities with education or employment (Blake-Holmes 2020). Perhaps this is particularly the case with instrumental care tasks. For instance, such practical difficulties could manifest when time conflicts arise because medical appointments or offices’ opening hours are during the time when they must be at school or work.

Additional evidence for contextual differences was found in the exploratory analyses based on the benefit finding subscales. Benefit finding in terms of perceived inner strengths (dimension growth) solely showed associations of positive valence with mental well-being. Perceived benefits in terms of stronger empathy towards others (dimension empathy; e.g., sensitivity toward others’ needs, caring about others, compassionate), however, only showed a positive association with mental well-being in the form of a direct path on mental well-being. The indirect paths were solely negative, as there was an indirect association over perceived helplessness, but no association over subjective coping. These exploratory findings appear to be important since feeling more empathetic with others is a previously described characteristic of young carers (e.g., Stamatopoulos 2018; Wepf et al. 2021) and will therefore need further attention.

### Limitations, Strengths, and Directions for Future Research

The main limitation of this study is the cross-sectional data. As benefit finding refers to past stressors, it seems plausible that it potentially predicts adolescents’ general sense of coping abilities and their thoughts and feelings in day-to-day life during the past month (perceived helplessness) or past two weeks (mental well-being), depending on how the questions were framed. Nevertheless, since all variables were assessed at the same point in time, causality or direction cannot be assumed. In addition, the definition of young carers in this study referred to a time frame of six months and therefore there is some uncertainty regarding how much caregiving would have taken place within the timeframes that the other variables were assessed in.

Another issue that deserves consideration refers to other possible indirect and context effects that were not addressed in this study. The direct effects of benefit finding on mental well-being in our study were more substantial than the indirect effects and therefore additional mechanisms may help to explain the association. For instance, benefit finding could lead to deeper relationships and trust in others, which in turn improve social support in stressful situations and thus impact mental well-being. The importance of social support for young carers’ well-being has been highlighted repeatedly, and benefit finding has been linked to this external coping resource (e.g., Pakenham and Cox 2018). Through benefit finding, young carers might see their caring roles in a positive light, and thus they openly talk about it in school among peers, with teachers, and other people in contact with them. On the one hand, an environment aware of young carers’ situations is more likely to offer support and assistance when needed to cope with difficulties. On the other hand, an accepting and open attitude towards their caring roles may also help young carers feel comfortable with themselves and others, which goes along with more support seeking and better mental well-being. These additional indirect effects (over social connectedness and openness about the caring role) and other potential mechanisms could be addressed in future studies.

A major strength of the study is reflected by the selection of instruments. First, the multidimensional measurement of benefit finding proved useful for the research questions in this study. Future studies should further examine the different dimensions of benefit finding and their impact on young carers’ positive mental health and development. Second, this study deliberately applied analyses and measures of benefit finding, coping, stress, and well-being which were not linked to the caring role specifically. This approach allowed for capturing the range of experiences that adolescents encounter in their many life domains. The flipside is that it limits the interpretation as the results only allow for speculation of which aspects of their life adolescents had in mind during the survey. When examining the patterns observed in this study, future research should rely on more detailed methods and, for instance, ask youth to report how they are coping with caring situations as compared to other life domains separately. Additionally, physiological markers of stress, as combined with self-reports, can support the robustness of effects.

### Policy and Practice Implications

In most countries, the topic of youth providing care has received modest attention in research, policy, and practice (Leu and Becker 2017). Accordingly, many young carers remain unrecognized as such and they receive little support (e.g., Nap et al. 2020). The current study’s findings add to the scant body of theoretically driven basic research in the field of young carers research that is needed to advance the knowledge to inform policy and practice (Joseph et al. 2020). Hence, the insights of this study build an essential piece for the design and development of appropriate interventions and support services for young carers.

Benefit finding would seem a potential starting point for interventions. However, this study highlights that young carers may struggle with a complex interplay of positive and negative feelings and perhaps contradictory motivations. To promote their mental well-being, it is, therefore, important that young carers are provided with spaces where they can reflect on their roles’ positive and negative aspects (e.g., What do I value about my caring role and what not? How does this help or hinder me?) and that they receive assistance and support in case they wish for it. Reflecting on their situations can take place by various means (e.g., writing, talking, art) and in different settings including individual times of rest and respite (e.g., creative, or sportive leisure activities, journaling), therapeutic one-to-one conversations, or group activities and meetings.

Understanding which types of tasks may promote or hinder the positive outcome in young carers is essential for the design of support services targeted at young carers and their families. The findings imply that instrumental tasks may impede young carers’ capacity for resilience. Improving young carers’ health literacy may be one way to address the burden due to their instrumental tasks. Services that offer help with caring should make sure information is provided in a youth-friendly language and that it is easily accessible for them. Professionals could help young carers to attain the required knowledge to handle the caring situation, and to facilitate access to information that helps young carers to manage instrumental tasks (e.g., filling out forms for health and disability insurance, attending to appointments with health professionals and other services or organizing them, etc.) with less effort or allocate them to professional services (e.g., translation services, social services staff in hospitals).

## Conclusion

Caring for a family member or close friend can be stressful for adolescents, but it may also provide an opportunity to grow as a person. A better understanding of the circumstances and ways that enable positive outcomes is essential to address young carers’ needs for support and recognition. Prior research indicated that young carers’ perceptions of benefits from caring lead to better adjustment. However, it remained unclear as to how and when benefit finding is associated with mental well-being in young carers. Therefore, this current study addressed potential pathways from benefit finding to mental well-being in young carers and examined whether the impact of benefit finding differs by the varying caring tasks young carers may be involved in. The findings support the assumption that benefit finding could be associated with mental well-being partly because of better subjective coping which in turn lowers feelings of helplessness. The results further suggest that the association between benefit finding and coping may specifically appear in the context where young carers are substantially involved in social and emotional caring tasks. In addition, this study indicated that more research is needed to unravel the complex associations between benefit finding and its dimensions with perceptions of stress. Nevertheless, the provided evidence suggests that benefit finding could be a key resource that promotes coping skills and mental well-being in young carers. By approaching benefit finding as the tendency of adolescents to derive benefits from past stressors in a general sense, this study opens new possibilities in research and practice that are relevant for young carers, but also for other groups of vulnerable youth.

## Footnotes

1 In the initial analysis plan, which was registered prior to the data collection (https://osf.io/gnrht), all PSS-10 items (i.e., across both subscales helplessness and self-efficacy) would have been used as indicators for one single mediator variable. However, the factorial structure implied two dimensions rather than one. Therefore, the hypothesis of benefit finding’s coping-enhancing and stress-buffering effect was modeled by the use of the PSS-10 perceived stress subscale and measures for subjective coping.

2 In a previous version of this manuscript, the multi-group analyses section also contained comparisons between young carers with high as compared to low caring in terms of time spent caring per week and perceived caring responsibility. These comparisons and variables were excluded to make the contribution more straightforward by focusing on one important dimension of the caring role, i.e., caring tasks.

## References

[CR1] Ali L, Ahlström BH, Krevers B, Skärsäter I (2012). Daily life for young adults who care for a person with mental illness: a qualitative study. Journal of Psychiatric and Mental Health Nursing.

[CR2] Blake-Holmes K (2020). Young adult carers: Making choices and managing relationships with a parent with a mental illness. Advances in Mental Health.

[CR3] Bodenmann, G. (2000). *Stress und Coping bei Paaren [Stress and coping among couples]*. Göttingen: Hogrefe Verl. für Psychologie.

[CR4] Bower JE, Moskowitz JT, Epel E (2009). Is benefit finding good for your health?. Current Directions in Psychological Science.

[CR5] Cassidy T (2013). Benefit finding through caring: the cancer caregiver experience. Psychology & Health.

[CR6] Cassidy T, Giles M (2013). Further exploration of the young carers perceived stress Scale: identifying a benefit-finding dimension. British Journal of Health Psychology.

[CR7] Cassidy T, Giles M, McLaughlin M (2014). Benefit finding and resilience in child caregivers. British Journal of Health Psychology.

[CR8] Cassidy T, McLaughlin M, Giles M (2014). Benefit finding in response to general life stress: measurement and correlates. Health Psychology and Behavioral Medicine.

[CR9] Cohen S, Kamarck T, Mermelstein R (1983). A global measure of perceived stress. Journal of Health and Social Behavior.

[CR10] Crane MF, Searle BJ, Kangas M, Nwiran Y (2019). How resilience is strengthened by exposure to stressors: the systematic self-reflection model of resilience strengthening. Anxiety, Stress, and Coping.

[CR11] Dharampal R, Ani C (2020). The emotional and mental health needs of young carers: What psychiatry can do. BJ Psych Bulletin.

[CR12] Folkman S (2008). The case for positive emotions in the stress process. Anxiety, Stress, and Coping.

[CR13] Greene J, Cohen D, Siskowski C, Toyinbo P (2017). The relationship between family caregiving and the mental health of emerging young adult caregivers. The Journal of Behavioral Health Services & Research.

[CR14] Haugland BSM, Hysing M, Sivertsen B (2019). The burden of care: a national survey on the prevalence, demographic characteristics and health problems among young adult carers attending higher education in Norway. Frontiers in Psychology.

[CR15] Helgeson VS, Reynolds KA, Tomich PL (2006). A meta-analytic review of benefit finding and growth. Journal of Consulting and Clinical Psychology.

[CR16] Hu L, Bentler PM (1999). Cutoff criteria for fit indexes in covariance structure analysis: conventional criteria versus new alternatives. Structural Equation Modeling.

[CR17] Ireland MJ, Pakenham KI (2010). The nature of youth care tasks in families experiencing chronic illness/disability: development of the youth activities of caregiving scale (YACS). Psychology & Health.

[CR18] Joseph S, Becker S, Becker F, Regel S (2009). Assessment of caring and its effects in young people: development of the multidimensional assessment of caring activities checklist (MACA-YC18) and the positive and negative outcomes of caring questionnaire (PANOC-YC20) for young carers. Child: Care, Health and Development.

[CR19] Joseph S, Sempik J, Leu A, Becker S (2020). Young carers research, practice and policy: an overview and critical perspective on possible future directions. Adolescent Research Review.

[CR20] Kallander EK, Weimand B, Ruud T, Becker S, van Roy B, Hanssen-Bauer K (2018). Outcomes for children who care for a parent with a severe illness or substance abuse. Child & Youth Services.

[CR21] Klein EM, Brähler E, Dreier M, Reinecke L, Müller KW, Schmutzer G (2016). The German version of the perceived stress scale—psychometric characteristics in a representative German community sample. BMC Psychiatry.

[CR22] Kline, R. B. (2016). *Principles and practice of structural equation modeling (methodology in the social sciences)*. New York: The Guilford Press.

[CR23] Landi, G., Pakenham, K. I., Crocetti, E., Grandi, S., & Tossani, E. (2021). Examination of the tripartite model of youth caregiving in the context of parental illness. *Psychology & Health.*10.1080/08870446.2020.1870116. 10.1080/08870446.2020.1870116 33417502

[CR24] Lang G, Bachinger A (2017). Validation of the German Warwick-Edinburgh mental well-being scale (WEMWBS) in a community-based sample of adults in Austria: a bi-factor modelling approach. Journal of Public Health.

[CR25] Lazarus, R. S., & Folkman, S. (1984). *Stress, appraisal, and coping*. New York: Springer.

[CR26] Leu A, Becker S (2017). A cross-national and comparative classification of in-country awareness and policy responses to ‘young carers’. Journal of Youth Studies.

[CR27] Leu, A., & Becker, S. (2019). Young carers. In H. Montgomery (Ed.), Oxford bibliographies in childhood studies. Oxford University Press. 10.1093/OBO/9780199791231-0120.

[CR28] McGibbon, M. (2021). The experiences of young carers in Northern Ireland: Negotiating pathways to a positive sense of self-identity—narratives of resilience, risk and identity. In L. Moran, K. Reilly, & B. Brady (Eds.), *Narrating childhood with children and young people (studies in childhood and youth)*. Cham: Palgrave Macmillan.

[CR29] Nap HH, Hoefman R, Jong N, de, Lovink L, Glimmerveen L, Lewis F (2020). The awareness, visibility and support for young carers across Europe: a Delphi study. BMC Health Services Research.

[CR30] Pakenham KI, Chiu J, Bursnall S, Cannon T (2007). Relations between social support, appraisal and coping and both positive and negative outcomes in young carers. Journal of Health Psychology.

[CR31] Pakenham KI, Cox SD (2018). Effects of benefit finding, social support and caregiving on youth adjustment in a parental illness context. Journal of Child and Family Studies.

[CR32] Preacher KJ, Hayes AF (2008). Asymptotic and resampling strategies for assessing and comparing indirect effects in multiple mediator models. Behavior Research Methods.

[CR33] Rosseel, Y. (2012). lavaan: an R package for structural equation modeling. *Journal of Statistical Software*. 10.18637/jss.v048.i02.

[CR34] RStudio Team. (2021). *RStudio: integrated development for R*. Bosten, MA.

[CR35] Skovdal M, Andreouli E (2011). Using identity and recognition as a framework to understand and promote the resilience of caregiving children in western Kenya. Journal of Social Policy.

[CR36] Smyth C, Blaxland M, Cass B (2011). ‘So that’s how I found out I was a young carer and that I actually had been a carer most of my life’. Identifying and supporting hidden young carers. Journal of Youth Studies.

[CR37] Stamatopoulos V (2018). The young carer penalty: exploring the costs of caregiving among a sample of Canadian youth. Child & Youth Services.

[CR38] Stewart-Brown S, Tennant A, Tennant R, Platt S, Parkinson J, Weich S (2009). Internal construct validity of the Warwick-Edinburgh mental well-being scale (WEMWBS): a Rasch analysis using data from the Scottish health education population survey. Health and quality of life outcomes.

[CR39] Svanberg E, Stott J, Spector A (2010). ‘Just helping’: children living with a parent with young onset dementia. Aging & Mental Health.

[CR40] Tennant R, Hiller L, Fishwick R, Platt S, Joseph S, Weich S (2007). The Warwick-Edinburgh mental well-being scale (WEMWBS): development and UK validation. Health and Quality of Life Outcomes.

[CR41] Wepf, H., Joseph, S., & Leu, A. (2021). Benefit finding moderates the relationship between young carer experiences and mental well-being. *Psychology & Health*. Advance online publication. 10.1080/08870446.2021.1941961. 10.1080/08870446.2021.1941961 34180332

